# Essential and Checkpoint Functions of Budding Yeast ATM and ATR during Meiotic Prophase Are Facilitated by Differential Phosphorylation of a Meiotic Adaptor Protein, Hop1

**DOI:** 10.1371/journal.pone.0134297

**Published:** 2015-07-30

**Authors:** Ana Penedos, Anthony L. Johnson, Emily Strong, Alastair S. Goldman, Jesús A. Carballo, Rita S. Cha

**Affiliations:** 1 Genome Damage & Stability Centre, University of Sussex, Brighton, BN1 9RQ, United Kingdom; 2 Division of Stem Cell Biology and Developmental Genetics, MRC National Institute for Medical Research, London, NW7 1AA, United Kingdom; 3 North West Cancer Research Fund Institute, School of Medical Sciences, Bangor University, Bangor, LL57 2UW, United Kingdom; 4 Department of Molecular Biology and Biotechnology, The University of Sheffield, Sheffield, S10 2TN, United Kingdom; National Cancer Institute, UNITED STATES

## Abstract

A hallmark of the conserved ATM/ATR signalling is its ability to mediate a wide range of functions utilizing only a limited number of adaptors and effector kinases. During meiosis, Tel1 and Mec1, the budding yeast ATM and ATR, respectively, rely on a meiotic adaptor protein Hop1, a 53BP1/Rad9 functional analog, and its associated kinase Mek1, a CHK2/Rad53-paralog, to mediate multiple functions: control of the formation and repair of programmed meiotic DNA double strand breaks, enforcement of inter-homolog bias, regulation of meiotic progression, and implementation of checkpoint responses. Here, we present evidence that the multi-functionality of the Tel1/Mec1-to-Hop1/Mek1 signalling depends on stepwise activation of Mek1 that is mediated by Tel1/Mec1 phosphorylation of two specific residues within Hop1: phosphorylation at the threonine 318 (T318) ensures the transient basal level Mek1 activation required for viable spore formation during unperturbed meiosis. Phosphorylation at the serine 298 (S298) promotes stable Hop1-Mek1 interaction on chromosomes following the initial phospho-T318 mediated Mek1 recruitment. In the absence of Dmc1, the phospho-S298 also promotes Mek1 hyper-activation necessary for implementing meiotic checkpoint arrest. Taking these observations together, we propose that the Hop1 phospho-T318 and phospho-S298 constitute key components of the Tel1/Mec1- based meiotic recombination surveillance (MRS) network and facilitate effective coupling of meiotic recombination and progression during both unperturbed and challenged meiosis.

## Introduction

Members of the conserved ATM/ATR family proteins are multi-functional serine/threonine kinases involved in a wide range of processes, including genome duplication, DNA damage repair, cell cycle progression, checkpoint regulation, and meiosis [1–3]. Meiosis is a specialized cell division program, during which a single round of genome duplication is followed by two successive rounds of genome segregation, resulting in halving of the genome. An essential feature of meiosis is that Spo11-catalyzed programmed meiotic DNA double strand breaks (DSBs) are converted to inter-homolog crossovers via meiotic recombination; the crossovers mediate accurate homolog disjunction during the first meiotic division or meiosis I (MI) [4]. During meiotic prophase, the ATM/ATR-based meiotic recombination surveillance (MRS) network ensures that cells do not undergo MI until all Spo11-DSBs are repaired [5, 6].

Central to ATM/ATR signalling is their phosphorylation of a class of proteins referred to as adaptors (or mediators): An adaptor is a scaffold protein that interacts with an effector kinase in an ATM/ATR phosphorylation dependent manner to activate the latter. An activated kinase in turn, phosphorylates relevant downstream targets that are necessary for a developmentally programmed cellular response [2, 7]. Evidence indicates that ATM/ATR utilization of an adaptor and/or effector kinase is regulated by the physiological state of the cell [7]. For example, in response to most forms of DNA damage, Tel1 and Mec1, the budding yeast ATM and ATR, utilize Rad9 (53BP1) and Rad53 (CHK2) as an adaptor and effector kinase, respectively [8, 9]. However, in response to replication stress, a different adaptor, Mrc1 (Claspin) is employed to activate Rad53 [10]. During meiosis, Tel1/Mec1 utilize Hop1, a conserved meiotic chromosome axis protein, and Mek1, a chromosome associated serine/threonine kinase, as a meiosis-specific adaptor and effector kinase, respectively [6, 11–13].

During meiotic prophase in budding yeast, where the molecular basis of ATM/ATR-function is best understood, Tel1 is activated by Spo11-catalysis of programmed DNA double strand breaks (DSBs) [14]; Mec1 activation, on the other hand, is dependent on single-stranded DNA and occurs following DSB resection [5]. Activated Tel1 and Mec1 phosphorylate a number of conserved meiotic proteins, including the above mentioned Hop1, Zip1, a component of the synaptonemal complex, and Rec114, a Spo11-accessory protein required for meiotic DSB formation [6, 15, 16].

An essential meiotic function of Tel1/Mec1 is to promote inter-homolog bias in meiotic recombination [6]. They achieve this via Hop1 phosphorylation, leading to phospho-Hop1-dependent activation of Mek1 [6]. Activated Mek1, in turn, is proposed to phosphorylate relevant target proteins, including Rad54, to ensure the inter-homolog bias in meiotic DSB repair [17, 18]. Another key function of Tel1/Mec1 is to mediate meiotic checkpoint responses. For example, they trigger meiotic arrest in response to accumulation of unrepaired meiotic DSBs in the absence of Dmc1, a conserved meiotic RecA protein [5, 19]. Intriguingly, Tel1 and Mec1 utilize the same adaptor and effector kinase, Hop1 and Mek1, respectively, for promoting the essential inter-homolog bias as well as for implementing meiotic checkpoint arrest [6].

Here we investigated the molecular basis of Tel1/Mec1-dependent signalling cascade mediated by Hop1/Mek1, allowing us to separate essential and checkpoint functions. We present evidence that the dual functionality is facilitated by differential phosphorylation of their meiotic adaptor Hop1 and the phosphorylation-dependent regulation of Mek1 activation.

## Results

### Hop1-S298 is an *in vivo* phosphorylation site

**Fig 1:**
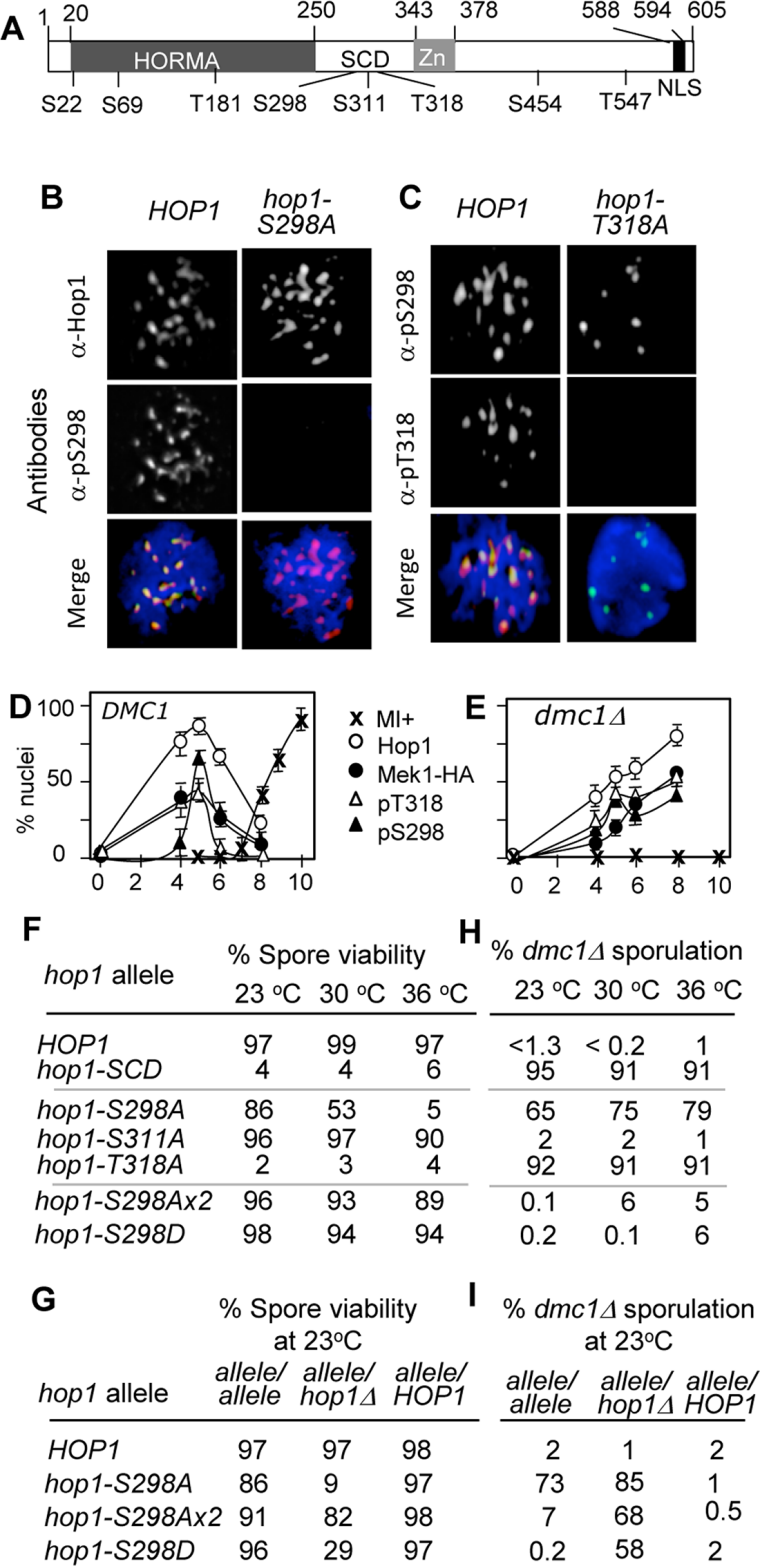
Lack of the Hop1-phospho-S298 leads to temperature- and dose- dependent meiotic failure. (A) Schematic representation of Hop1 with the locations of eight [S/T]Q motifs. S: serine, T: threonine, SCD: [S/T]Q Cluster Domain. Also shown are the HORMA domain, Zn finger motif, and nuclear localization signal (NLS). (B) and (C) Specificity of the phospho-specific α-pS298 and α-pT318 antibodies. Nuclear spreads of *HOP1* and *hop1-S298A* panel (B) or *HOP1* and *hop1-T318A* panel (C) were prepared from samples taken at 5hours after induction of synchronous meiosis at 23°C. The spreads were stained with DAPI and the antibodies against either the phospho-S298 panel (B) or the phospho-T318 panel (C). (D) and (E). *In vivo* phosphorylation of Hop1-S298 and T318 during *DMC1* (D) or *dmc1Δ* (E) meiosis at 23°C. Nuclear spreads of a *DMC1* or *dmc1Δ* strain were prepared from samples collected at the indicated time points. The spreads were stained with the antibodies against Hop1, HA (for detection of Mek1-HA), and the two phospho-specific antibodies, α-pS298 and α-pT318. A nucleus exhibiting 10 or more foci of each epitope was scored as a positive. Also shown are the fractions of cells having undergone first meiotic division or meiosis I (MI) at each time point. Errors were calculated from the 95% confidence interval of a binomial distribution. (F) Spore viability of homozygous diploid strains of indicated genotype at specified temperature. For each genotype, at least 80 spores were analysed. A: Alanine; D: aspartic acid, *hop1-S298Ax2*: an allele containing two tandem copies of *hop1-S298A*. *hop1-SCD*: an allele where the S298, S311, and T318 in the SCD are mutated to A [6]. *hop1-S311A*: an allele where the S311 is mutated to A. (G) Spore viability of indicated *HOP1* alleles at 23°C as a either homozygous diploid (*allele/allele*), hemizygous diploid (*allele*/ *hop1Δ*) or heterozygous diploid (*allele/HOP1*). (H) Sporulation efficiency of *dmc1Δ* strains in the indicated *hop1* mutation background. (I). Sporulation efficiency of *dmc1Δ* strains in the indicated *hop1* mutation background at 23°C as a either homozygous diploid (*allele/allele*), hemizygous diploid (*allele*/ *hop1Δ*) or heterozygous diploid (*allele/HOP1*).

Hop1 contains eight ATM/ATR consensus sites (nine in the SK1 strain background), referred to as SQ/TQ motifs, each comprising of a serine (S) or threonine (T) followed by a glutamine (Q) (Fig 1A). Of the eight SQ/TQ motifs, the phospho-T318 is required for the essential recruitment and activation of Mek1, while the threonine at position 181 might play a different role [6]. When replacing any of the remaining SQ/TQ sites to alanine, a residue that cannot be phosphorylated, all of the mutant alleles appear to behave indistinguishably from the wild type during unchallenged meiosis, except for the serine 298 (S298), elimination of which confers a modest reduction in spore viability [6] (below).

To confirm that the Hop1-pS298 was an *in vivo* phosphorylation site, we generated antibodies against the corresponding phospho-peptide, referred to as α-pS298 (Materials and Methods). As a control, we also raised antibodies against a confirmed *in vivo* phospho-residue, the Hop1 phospho-T318, referred to as α-pT318 [6, 20]. Cytological analysis showed that both the α-pS298 and α-pT318 antibodies generated signals in nuclear spread samples prepared from a WT control and that these signals co-localized with α-Hop1 foci (Fig 1B and 1C). Importantly, the α-pS298 antibodies did not generate any signals in a strain expressing a mutant allele, *hop1-S298A*, where the corresponding S298 was replaced with a non-phosphorylatable alanine (A) (Fig 1B; S1A and S1B Fig). Similarly, the α-pT318 antibodies did not generate a signal in a *hop1-T318A* background, where the T318 was replaced with an alanine residue (Fig 1C; S1A and S1B Fig).

The Hop1 phospho-S298 or phospho-T318 signals were observed transiently during meiotic prophase (Fig 1D), the period during which Hop1 is known to undergo transient Tel1/Mec1-dependent phosphorylation [6, 21]. In a *dmc1Δ* background, Hop1 phosphorylation does not turn over but is maintained in a Tel1/Mec1-dependent manner [6, 22]. We observed that the α-pT318 and α-pS298 signals in a *dmc1Δ* background did not turn over either, but continued to accumulate (Fig 1E). These observations taken together, we conclude that the Hop1-S298 is an *in vivo* Tel1/Mec1 phosphorylation site, which becomes phosphorylated during both normal and challenged meiosis.

### Prevention of Hop1 phosphorylation at Ser298 confers a dose- and temperature-dependent meiotic failure

Having confirmed *in vivo* phosphorylation of the Hop1-S298, we proceeded to investigate its function(s). To this end, we characterized the above mentioned non-phosphorylatable allele, *hop1-S298A*. Spore viability of a *hop1-S298A* strain was temperature-sensitive in that it dropped from 86% at 23°C to 5% at 36°C (Fig 1F; S1C Fig). In contrast, spore viability of the other *hop1* alleles tested (i.e. *hop1-SCD*, *hop1-S311A*, and *hop1-T318A)* was unaffected by changes in temperature (Fig 1F). A strain expressing a phospho-mimetic allele, *hop1-S298D*, where the S298 was replaced with a negatively charged aspartic acid residue (D) was viable at all temperatures (Fig 1F). Doubling copy number of the *hop1-S298A* also improved spore viability at 36°C from 5% to 89% (Fig 1F, *hop1-S298Ax2*), while halving it reduced the viability at 23°C from 86% to 9% (Fig 1G, compare *allele/allele* and *allele/hop1Δ* for *hop1-S298A*).

The temperature- and dose-dependent spore viability of a *hop1-S298A* strain suggested that the phospho-S298 might be required for Hop1 stability at high temperature. However, analysis showed that neither the mutation nor temperature caused substantial reductions in Hop1 levels, relative to wild type (S1D Fig). We also found that Hop1 chromosome association was normal in a *hop1-S298A* background at high temperature (data not shown).

### Phosphorylation of Hop1 at S298 is required for preventing *DMC1*-independent DSB repair

Inactivation of Dmc1 triggers Mec1-mediated meiotic arrest, which is dependent on the Hop1 phospho-T318 [5, 6]. To test whether the phospho-S298 was similarly required, we assessed sporulation efficiency of a *hop1-S298A dmc1Δ* strain. Results showed that the double mutant sporulated efficiently, with its sporulation efficiency ranging from 65% at 23°C to 79% at 36°C (Fig 1H). On the other hand, expression of the phospho-mimetic allele *hop1-S298D* or multi-copy *hop1-S298Ax2* restored the arrest (Fig 1H and 1I). We conclude that the phospho-S298, like the phospho-T318, is required for implementing *dmc1Δ* arrest.

**Fig 2:**
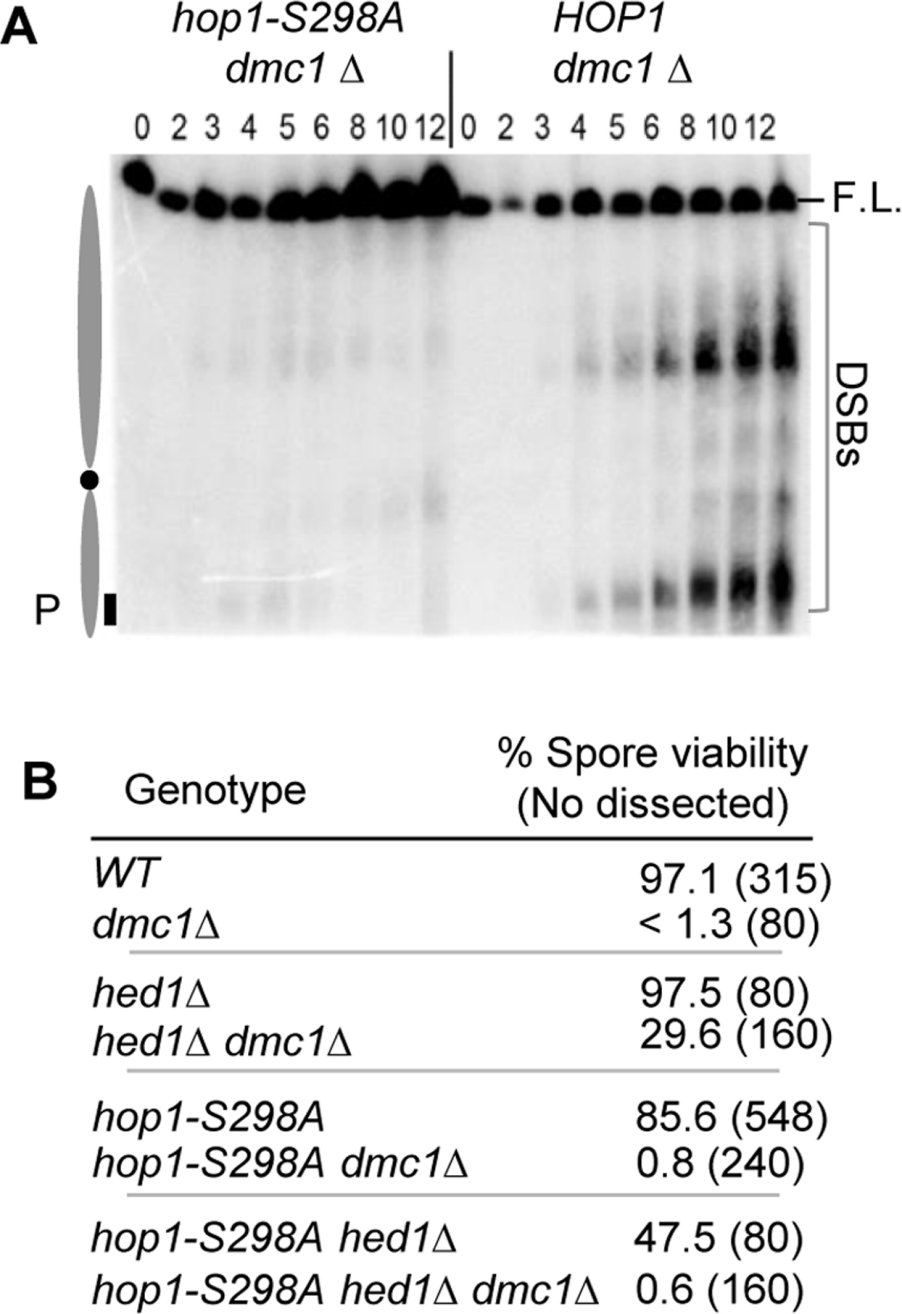
Genetic interaction among *hop1-S298A*, *dmc1Δ*, and *hed1Δ*. **A**. PFGE/Southern analysis of ChrIII was performed on samples prepared from a *hop1-S298A dmc1Δ* or *HOP1 dmc1Δ* strain. Positions of the full length (FL) and DSBs are indicated on the right side of the gel. Positions of the *CHA1* probe (P) and centromere (filled circle) are indicated on the left side of the gel. **B**. Spore viability of homozygous diploid strains of the indicated genotypes at 23°C. For each genotype, at least 80 spores were analysed.

*dmc1Δ*-mediated meiotic arrest is triggered by accumulation of unrepaired meiotic DSBs, which activates the checkpoint function of Tel1/Mec1 [19]. The arrest can be bypassed by either the mutations that permit meiotic progression in the presence of unrepaired breaks (e.g. *mec1-1*, *rad24Δ*, or *rad17Δ*) or allowing Rad51 mediated DSB repair (e.g. *hed1Δ*, *hop1-T318A* or *mek1Δ*) [5, 6, 22–24]. Rad51 is the other budding yeast RecA homolog, whose recombinase function becomes inhibited during meiosis by its meiosis-specific inhibitor Hed1 [24, 25]. To test which of the two mechanisms was responsible for the *hop1-S298A* alleviation of *dmc1Δ* meiotic arrest, we examined the status of meiotic DSBs in a *hop1-S298A dmc1Δ* strain. Results showed that breaks did not accumulate in the double mutant (Fig 2A). Since Spo11 catalysis initiates normally in the absence of the Hop1 phospho-S298 [6], the latter suggests that the *hop1-S298A* alleviation of *dmc1Δ* arrest is attributable to Rad51-mediated recombination, circumventing accumulation of unrepaired DSBs.

### Hop1-S298 phosphorylation supports high levels of spore viability in the absence of *HED1*

High spore viability of a *hop1-S298A* strain at 23°C (Fig 1F, 86%) implies that the phospho-S298 can be dispensable for essential crossover formation under certain conditions. The latter, in turn, raises the possibility that the *DMC1*-independent break repair in a *hop1-S298A dmc1Δ* strain at 23°C might proceed with inter-homolog bias and restore spore viability of a *dmc1Δ* strain. We tested this possibility and found that spore viability of a *hop1-S298A dmc1Δ* strain was very low (0.8%; Fig 2B). We conclude that DSB repair in a *hop1-S298A dmc1Δ* background does not proceed with inter-homolog bias.

Deletion of *HED1*, the gene encoding for a meiosis-specific inhibitor of Rad51, restores spore viability of *dmc1Δ* cells, indicating that Rad51-mediated DSB repair in a *hed1Δ dmc1Δ* background can proceed with reduced inter-homolog bias [24, 26]. We observed that *hop1-S298A* led to a significant reduction in spore viability of a *hed1Δ dmc1Δ* strain, from 29.6% to 0.6% (Fig 2B). Therefore, the residual inter-homolog bias in Rad51-mediated recombination in a *hed1Δ dmc1Δ* background is dependent on the Hop1 phospho-S298. Furthermore, we observed synthetic interaction between *hop1-S298A* and *hed1Δ*even in the presence of Dmc1, with the spore viability of a *hed1Δ hop1-S298A DMC1* strain (47.5%) being notably reduced at 23°C compared to either *hed1Δ DMC1* (97.5%) or *hop1-S298A DMC1* (86%) (Fig 2B).

### Hop1-S298 phosphorylation is dispensable for essential Mek1 activation during normal meiosis

**Fig 3:**
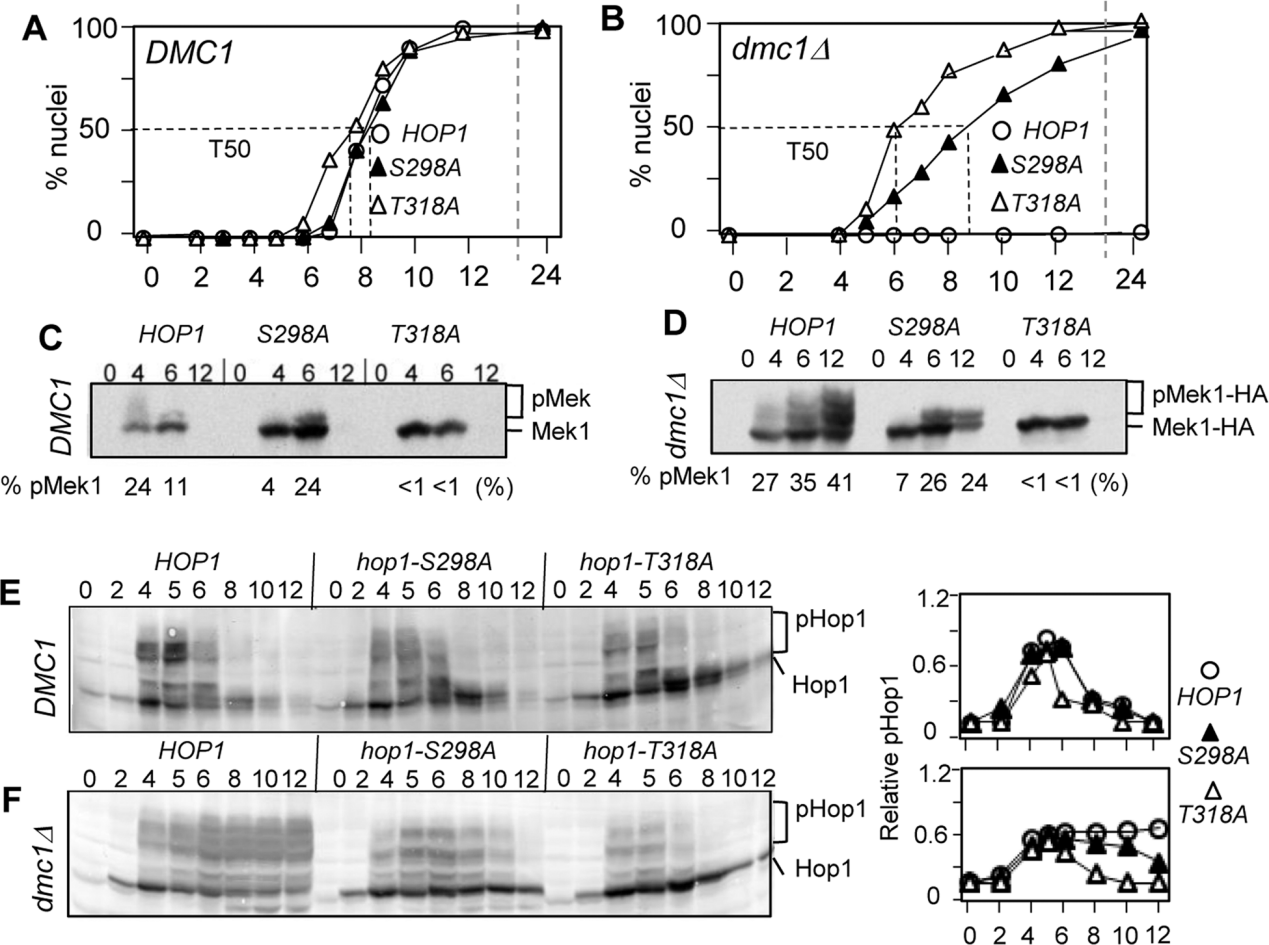
Hop1-S298 phosphorylation is required for *dmc1Δ*-dependent meiotic checkpoint response. (A) and (B). Fraction of cells undergone meiosis I (MI) in synchronous meiotic cultures of *HOP1*, *hop1-S298A*, and *hop1-T318A* at 23°C in a *DMC1* or *dmc1Δ* background. T50: Time at which 50% of the active culture has completed MI. At least three separate timecourses were considered, for each set of strains for each background. T50 kinetics were calculated from the most representative timecourse for each set of strain and *DMC1/dmc1* background. Errors were calculated from the 95% confidence interval of a binomial distribution. (C) and (D). Effects of *hop1-S298A* and *hop1-T318A* on Mek1-HA phosphorylation during *DMC1* and *dmc1Δ* meiosis. Samples from the cultures described in panels (A) and (B) were subjected to Western blot analysis using anti-HA antibody. Positions of the unphosphorylated and phosphorylated Mek1-HA species are indicated to the right of the blot. ‘% pMek’ corresponds to the proportion of phosphorylated Mek1-HA species in each lane calculated by dividing the signal found in the ‘pMek1-HA’ area of the gel by the total signal (‘pMek1-HA’ + ‘Mek1-HA’). (E) and (F). Effects of *hop1-S298A* and *hop1-T318A* on Hop1 phosphorylation during *DMC1* and *dmc1Δ* meiosis. Samples from the cultures described in panels (A) and (B) were subjected to Western blot analysis using anti-Hop1 antibody. Positions of the unphosphorylated and phosphorylated Hop1-species are indicated to the right of the blot. Shown on the right panels is quantification analysis of the Western images, where the signal in the ‘pHop1’ region in each lane is divided by the total signal (‘pHop1’+ ‘Hop1’) in the corresponding lane.

Genetic evidence above suggests that the Hop1 phospho-S298 plays an auxiliary role, along with the essential phosho-T318, to promote spore viability and mediate *dmc1Δ* meiotic arrest. We wished to address the molecular basis of its function. Since an essential function of the Tel1/Mec1 phosphorylation of Hop1 is to activate Mek1 [6], we proceeded to assess the effects of *hop1-S298A* on Mek1 phosphorylation. In a *HOP1* strain during normal meiosis, Mek1 phosphorylation was modest and transient, observed at 4 and 6 hours (Fig 3C). Comparable levels of Mek1 phosphorylation, reaching ~24% of total Mek1-HA signal at t = 6 hours, were observed in *hop1-S298A* cells (Fig 3C). As shown previously [6, 20], no Mek1 activation was observed in a *hop1-T318A* background. We conclude that the Hop1 phospho-S298, unlike the phospho-T318, is dispensable for the essential Mek1 activation during unchallenged meiosis.

### Hop1-S298 phosphorylation promotes hyper-phosphorylation of Mek1 necessary for mediating *dmc1Δ* meiotic arrest

Deletion of *DMC1* leads to constitutive Mek1 phosphorylation and changes in its mobility shift-pattern that are indicative of an additional phosphorylation event(s) [6, 20] (Fig 3D). Constitutive Mek1 phosphorylation was also observed in a *hop1-S298A dmc1Δ*background; however, the pattern of Mek1 mobility shift in the latter remained comparable to *HOP1 DMC1* or *hop1-S298A DMC1*, revealing that the Mek1 hyper-phosphorylation in a *dmc1Δ* background is dependent on the phospho-S298.

*HOP1 dmc1Δ* cells arrest before the onset of first meiotic division or meiosis I (MI) (Fig 3B) [19]. In contrast, *hop1-S298A dmc1Δ* or *hop1-T318A dmc1Δ* cells underwent MI (Fig 3B). In a *hop1-S298A dmc1Δ* background, where the basal level Mek1 phosphorylation was maintained (Fig 3D), the onset of MI was delayed by approximately 1 hour (Fig 3A and 3B). In a *hop1-T318A* background, where Mek1 phosphorylation does not occur, no *dmc1Δ*-dependent delay was observed (Fig 3A and 3B). Similar correlation between the extent of Mek1-phosphorylation and robustness of *dmc1Δ*-induced meiotic arrest was observed among different *hop1-S298* alleles (i.e. *hop1-S298A*, *hop1-S298D*, and *hop1-S298Ax2*, Fig 1H and 1I) (S2 Fig). Taken together, we conclude that the Hop1 phospho-T318-dependent recruitment and activation of Mek1 is necessary but not sufficient to implement *dmc1Δ* meiotic arrest; the arrest requires further activation of Mek1 mediated by the Hop1 phospho-S298.

### Hop1 phospho-S298 ensures constitutive Hop1/Mek1-signalling in the absence of Dmc1

Following Spo11-catalysis, Hop1 is phosphorylated at multiple residues, including most, if not all, of the eight Tel1/Mec1-consensus sites [6, 20, 21]. We observed comparable levels of Hop1 phosphorylation among *HOP1*, *hop1-S298A*, and *hop1-T318A* strains in a *DMC1* background, indicating that neither the phospho-T318 nor the phospho-S298 have a significant effect on the transient Hop1 phosphorylation during unchallenged meiosis (Fig 3E). In contrast, the *dmc1Δ*-dependent constitutive Hop1 phosphorylation was impaired in both mutants (Fig 3F).

**Fig 4:**
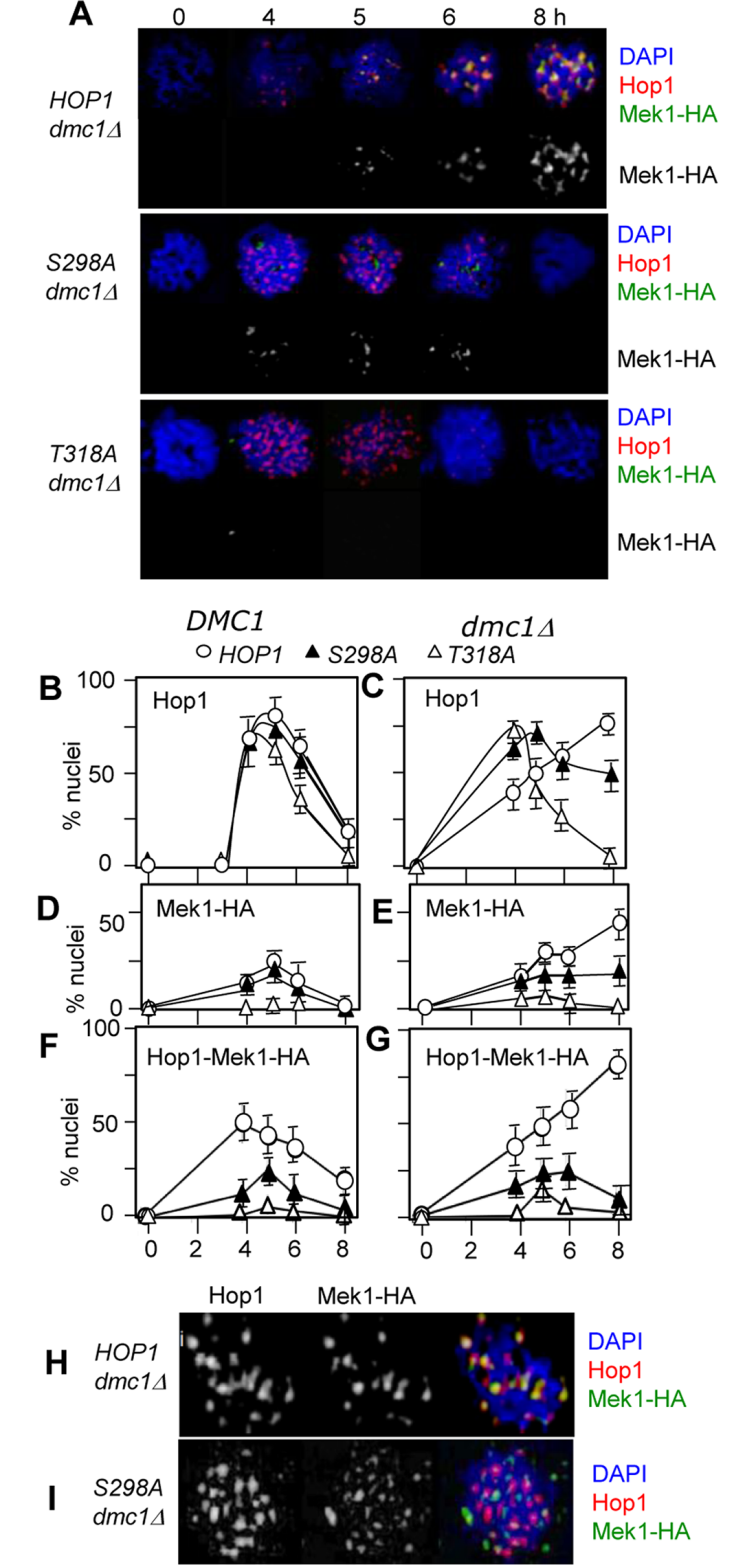
Hop1-S298 phosphorylation promotes stable Mek1-Hop1 interaction on chromosomes. (A) Hop1 and Mek1-HA chromosome association during *dmc1Δ* meiosis at 23°C in a *HOP1*, *hop1-S298A*, or *hop1-T318A* background. (B) and (C) Effects of *hop1-S298A* or *hop1-T318A* on Hop1 chromosome association during *DMC1* or *dmc1Δ* meiosis. Fraction of cells exhibiting 10 or more Hop1 foci was scored. (D) and (E) Effects of *hop1-S298A* or *hop1-T318A* on Mek1-HA chromosome association during *DMC1* or *dmc1Δ* meiosis. Fraction of cells exhibiting 10 or more Mek1-HA foci was scored. (F) and (G) Effects of *hop1-S298A* and *hop1-T318A* on Hop1-Mek1 co-localization during *DMC1* and *dmc1Δ* meiosis. Fraction of nuclei where more than 80% of Mek1-HA foci co-localized with Hop1 foci was scored. (B-G) Errors were calculated from the 95% confidence interval of a binomial distribution. (H) and (I) Effects of *hop1-S298A* on Hop1-Mek1 interaction on chromosomes. Nuclear spreads of *HOP1 dmc1Δ* and *hop1-S298A dmc1Δ* were prepared from samples taken at 6 hours after induction of synchronous meiosis at 23°C. The spreads were stained with DAPI and the antibodies against Hop1 and HA (for detection of Mek1-HA).

Next, we assessed the effects of *hop1-S298A* on Hop1- and Mek1- chromosome association. In a *DMC1* background, *hop1-T318A* cells exhibited a modest reduction in transient Hop1-chromosome association and no detectable signal in Mek1 association (Fig 4B and 4D). In a *hop1-S298A DMC1* background, both Hop1- and Mek1-chromosome association occurred normally (Fig 4B and 4D), supporting the observation above that the phospho-S298 is dispensable for the essential Mek1 activation during normal meiosis. In the absence of *DMC1*, the *dmc1Δ*-dependent maintenance of Hop1/Mek1 chromosome association was impaired in a *hop1-S298A* as well as a *hop1-T319A* background (Fig 4A, 4C and 4D).

### Hop1-phospho S298 promotes stable Mek1-Hop1 interaction on chromosomes

Potential effects of the Hop1 phospho-S298 on co-localization between Hop1 and Mek1 were assessed. In a *HOP1* background, nearly all Mek1 foci were found nearby a Hop1 signal (Fig 4H). In contrast, the majority of the Mek1 foci in a *hop1-S298A* background was found without a nearby Hop1 signal (Fig 4I). Quantitative analysis revealed that the fraction of nuclei exhibiting significant co-localization between Hop1 and Mek1 was significantly reduced in a *hop1-S298A* background, irrespective of the status of *DMC1* (Fig 4F and 4G). We conclude that the Hop1 phospho-S298 promotes the continued Hop1-Mek1 interaction on chromosomes following the initial phospho-T318 mediated recruitment of Mek1.

## Discussion

### Hop1 phospho-T318 and phospho-S298 mediate gradual activation of Hop1 and Mek1

Taken together, evidence thus far suggests that the Tel1/Mec1 activation of Hop1/Mek1 during normal or challenged meiosis occurs in a gradual manner dependent on the Hop1 phospho-T318 and the phospho-S298: (i) For the transient Hop1 phosphorylation during normal meiotic prophase I, neither the phospho-T318 nor the phospho-S298 is required. (ii) For the essential Mek1 recruitment and activation during normal meiosis, only the phospho-T318 is required. And (iii) For the Mek1 hyper-phosphorylation and constitutive Hop1/Mek1 chromosome-association necessary for implementing *dmc1Δ* meiotic arrest, both the phospho-T318 and the phospho-S298 are required.

We first showed the requirement for the phosphorylation of Hop1 at the SCD on Mek1 recruitment to the axial elements and for its activity [6]. Furthermore, we showed that among the three S/T[Q]s present at the SCD in Hop1, only the phospho-T318 was strictly essential for Hop1 activity over Mek1 initial recruitment and function [6, 20]. We and others proposed that the phospho-dependent activation of Mek1 through phospho-Thr318 relied on its possible interaction with the forkhead-associated (FHA) domain present in Mek1. FHA domains are modular phosphopeptide recognition domains with a striking specificity for phosphorylated threonine residues in target proteins [27–29]. Replacing p-Thr by p-Ser severely compromises binding and the associated function triggered by the FHA-phosphopeptide complex formation [6, 30]. Whereas the presence of a phospho-threonine is still a requirement, some studies have shown that the situation might be more complicated than originally thought, for example, combinations of mono- bi- or tri- phospho-peptide can display differential binding affinity to certain FHA modules *in vitro* [20, 31, 32].

Interestingly, multi-phospho-peptide binding to FHA modules has been proposed as the mechanism that could induce hierarchical co-operative association of two FHA modules in a sequential manner. This is manifested in proteins that contain segments with multiple phosphorylated threonines as well as serines [31, 32]. Here we present evidence of the existence of synergistic function for one of the two T318 neighbouring phospho-serines, part of the SCD. p-Ser298 is a confirmed phosphorylation site that could enhance or mediate, signal amplification under conditions where persistent and robust, Mek1 association with Hop1, like it occurs in *dmc1* mutants, is required. Interestingly, p-Ser298 is surrounded by three other threonines in position -1, +2 and +3, which could also be subjected to phosphorylation, perhaps by a kinase activity promoted by the initial binding of Hop1-pThr318 with FHA-Mek1, and triggered by the presence of phospho-serine298. It is tempting to speculate that phosphorylation within the SCD of Hop1 of additional threonines near p-Ser298 could serve as secondary binding platforms for another FHA-module for a different Mek1 molecule, thus favouring oligomerization and trans-activation of Mek1 [21]. Although several other scenarios might be possible [21, 22, 33–36], the need for more genetic, biochemical and structural data, together with the identification of additional posttranslational modifications in Hop1, Red1 and Mek1 should be the subject of future studies to help identifying the mechanism underlying Mek1 activation.

Another important clue emerging from this study is the confirmation for the need of multiple phosphorylation sites in the context of two interacting molecules during the response to meiotic DSBs. Most ATR/ATM targets, with many of them usually involved in multi-complex formation triggered by DNA damage, contain clusters of S/T[Q]s (SCDs) as opposed to a single reactive phospho-residue [37]. Specific subsets of phosphorylations in Hop1 might select for specific activities in this multi-functional adaptor protein.

Currently, the basis of the phospho-T318-independent Mek1 chromosome-association remains unknown. It is possible that Mek1 is recruited to chromosomes via Red1, another meiotic chromosome axis protein known to form a complex with both Hop1 and Mek1 [13, 38, 39].

### Model: Hop1 phospho-T318- and -S298-dependent stepwise activation of Mek1 facilitates Tel1/Mec1-dependent coupling of meiotic recombination and progression

**Fig 5:**
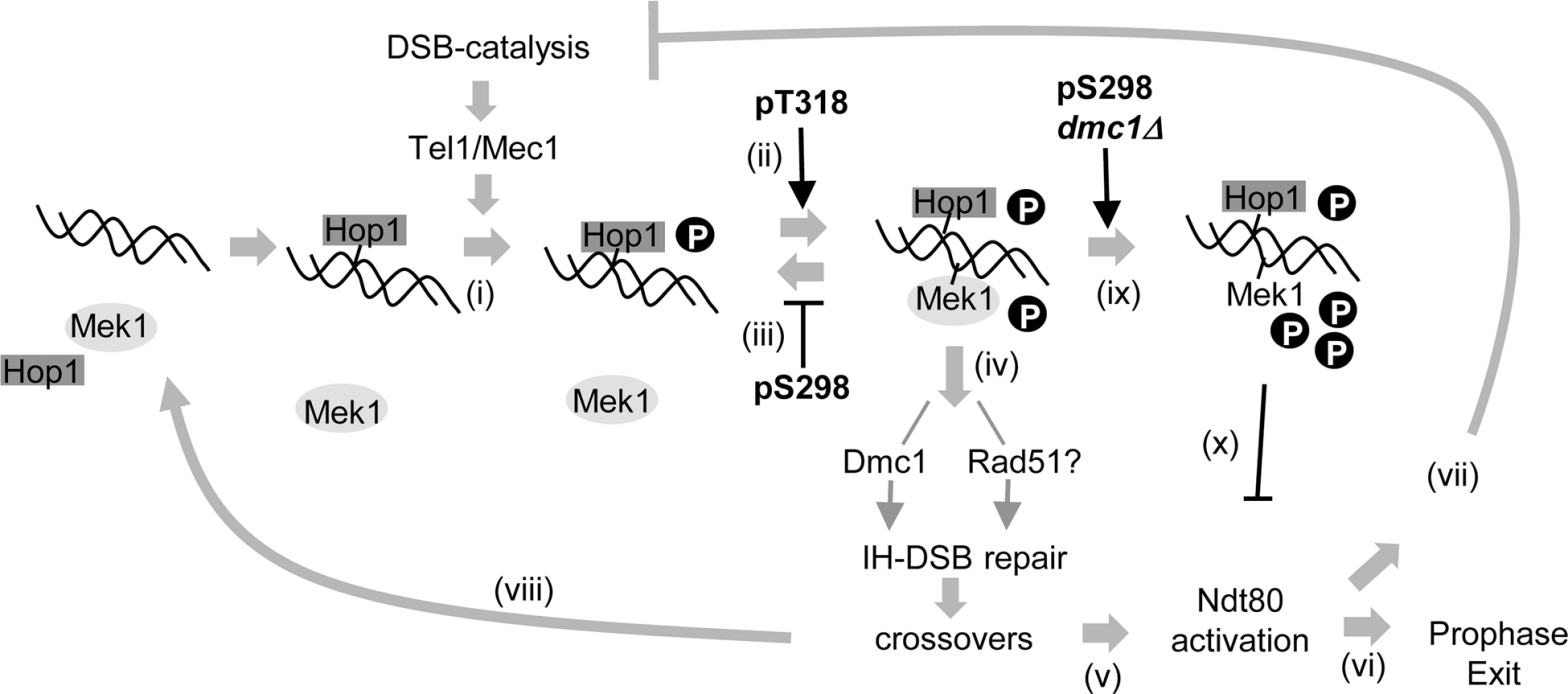
Model: Tel1/Mec1 phosphorylation of Hop1 at the T318 and S298 ensures effective coupling of meiotic recombination and progression. (i) Spo11-catalysis of meiotic DSBs triggers Tel1/Mec1 phosphorylation of chromosome bound Hop1 at multiple residues, including the T318 and S298. (ii) The phospho-T318 mediates the initial Mek1-recruitment and phosphorylation, independently of the phospho-S298. (iii) The phospho-S298 promotes stable Hop1-Mek1 interaction on chromosomes. (iv) The phospho-T318 and phospho-S298 promote spore viability by ensuring inter-homolog repair of meiotic breaks; available genetic evidence suggests that the phospho-T318 and phospho-S298 might be involved in regulating the Dmc1- and Rad51-dependent repair process, respectively (see text). (v) Once the essential crossover requirement is met, Ndt80 is activated, leading to exit from meiotic prophase (vi) and irreversible inactivation of Spo11-complex (vi). (viii) Hop1/Mek1 de-phosphorylation and removal from chromosomes ensue, accounting for the transient activation of the Hop1/Mek1-signalling during unchallenged meiosis. (ix, x) During challenged meiosis (e.g. *dmc1Δ)*, Mek1 undergoes the Hop1 phospho-S298-dependent hyper-phosphorylation (ix), necessary for implementing a meiotic checkpoint response (x).

The evidence shown above indicates that the Tel1/Mec1 activation of Hop1/Mek1 proceeds in a stepwise manner dependent on the Hop1 phospho-T318 and phospho-S298: The phospho-T318 mediates essential Mek1 recruitment and phosphorylation (Fig 5ii) and the phospho-S298 promotes stable interaction between Hop1 and Mek1 on chromosomes, following the phospho-T318-dependent Mek1 recruitment (Fig 5iii). While both phospho-T318 and -S298 contribute to an essential function(s) of Hop1, our findings suggest that contribution of the phospho-S298 is minor compared to the essential Hop1 phospho-T318.

What could be the role of the phospho-S298? The observed synthetic interaction between *hed1Δ* and *hop1-S298A* suggests that the phospho-S298 might have a role in regulating Rad51 activity. For instance, in the absence of Hed1, the phospho-S298 might assume the role of Hed1 and inhibit Rad51-mediated DSB repair. However, the fact that the phospho-S298 is required for viability of *hed1Δ dmc1Δ* spores (above) would argue against the notion that the phosphorylation prevents Rad51-mediated recombination. Instead, the Hop1 phospho-S298 might be involved in ensuring inter-homolog bias of Rad51-mediated DSB repair in *hed1Δ*. An implication of the latter would be that Rad51-mediated meiotic recombination, similar to the Dmc1-mediated process, is subjected to regulatory process that promotes inter-homolog bias. It is tempting to speculate that the Hop1 phospho-T318 and phospho-S298 might mediate essential crossover formation by regulating the Dmc1- and Rad51-mediated repair pathways, respectively (Fig 5iv).

Earlier works have shown that Mek1 can phosphorylate other targets which might impact in the outcome of Rad51 strand invasion activity. Rad54, a dsDNA-dependent ATPase, facilitates homologous recombination in concert with Rad51. Phosphorylation of Rad54 by Mek1 attenuates its interaction with Rad51 as well as reducing Rad51 activity [17]. The possibility that Hop1-pS298 could be required to promote this activity might seem obvious, nevertheless, we cannot exclude other more complex scenarios where Rad54 inhibition would not be essential to reinforce IH-bias, for example by Mec1/Hop1-pS298-dependent regulation of the other dsDNA-dependent ATPase, Tid1/Rdh54 [40].

Evidence suggests that the Tel1/Mec1-control of meiotic progression is via Ndt80 activation [15, 41]. Ndt80 is a meiotic transcription factor required for exit from meiotic prophase (Fig 5vi) and irreversible inactivation of the Spo11-complex (Fig 5vii) [15, 42, 43]. Interestingly, we observed that the Hop1 phopho-S298 was required for spore viability of a mutant with reduced Spo11-catalysis (*rec114-8D*) [15], which suggests that the phospho-S298 might also contribute to viable spore formation by preventing premature inactivation of the Spo11-complex until the requirement for essential crossover formation is satisfied.

During normal meiosis, cells would eventually acquire a sufficient level of crossovers and exit meiotic prophase (Fig 5v and 5vi). Hop1/Mek1 dephosphorylation and removal from chromosomes would ensue, accounting for the transient nature of Hop1/Mek1 activation (Fig 5viii). In the absence of Dmc1, meiotic DSBs accumulate and trigger a Tel1/Mec1- and Hop1/Mek1-dependent meiotic arrest. Here, we demonstrate that the arrest is dependent on the Hop1 phospho-S298-mediated Mek1 hyper-phosphorylation (Fig 5ix and 5x). Currently, the nature of the phospho-S298 and *dmc1Δ*-dependent Mek1 phosphorylation remains unknown. Notably however, we observed a synthetic interaction between *hop1-S298A* and *mek1-S320A*, a *mek1* allele lacking a phosphorylation site required for mediating *dmc1Δ* arrest, suggesting an involvement of the Mek1 phospho-S320 [21, 22] (S3 Fig).

In summary, evidence presented above indicates that the Tel1/Mec1 activation of Hop1/Mek1 during meiotic prophase proceeds in a stepwise manner dependent on Hop1 phospho-T318, phospho-S298, and the status of meiotic recombination. We propose that the phospho-T318 and phospho-S298 constitute key components of the Tel1/Mec1-based meiotic recombination surveillance (MRS) network [15, 44, 45] and that they ensure a successful meiotic outcome during both normal and challenged meiosis by facilitating effective coupling of meiotic recombination and progression.

## Materials and Methods

### Yeast manipulation

All strains were diploids of the SK1 background; relevant genotypes of the strains are listed in S1 Table. Mutagenesis of *HOP1* containing plasmid and integration in *hop1Δ* strains was performed as in [6]. Integration and copy number were confirmed by digesting DNA from transformed colonies with the restriction enzyme BamHI. Southern blots were then performed where membranes were hybridized using a probe that mapped within the *URA3* ORF. Correct integration of a single copy appeared as two bands of approximately14kbp and 6kpb. Multiple integrations appeared as a third band of ~8.4kbp. Additional number of copies of Hop1 plasmids (8.4kbp) were estimated by quantifying the intensity of the third band and was then compared it with the intensities of the 14kbp and the 6kbp bands. *hop1-S298Ax2* was considered when the intensity of the 8.4kbp band was approximately equivalent in intensity to each of the other two individual bands (14kbp and 6kbp). Induction of synchronous meiosis was carried out according to a described protocol [16]. All pre-growth was carried out at 30°C; meiotic time courses were carried out at 23°C, 30°C, or 33°C as indicated.

### Generation of phospho-specific Hop1 antibodies

Polyclonal antibodies against the Hop1 phospho-T318 and phospho-S298 were obtained as following: The α-pT318 polyclonal antibody [Cambridge Research Biochemicals] was obtained by immunising two rabbits with the antigenic[C]-Ahx-ASIQP-[pT]-QFVSN where C represents the C-terminus of the peptide, Ahx is aminohexanoicacid and pT is a phosphorylated threonine residue. Upon bleeding, antibodies were purified through two affinity columns (each followed by a purification pass), the first adsorbing antibodies that bind to non-phosphorylated peptides and the second adsorbing the phospho-specific antibodies to pT318. The specificity of the antibody was tested using ELISA (enzyme-linked immunosorbent assay) analysis. The polyclonal phospho-specific antibody against phosphorylated serine residue 298 [Eurogentec] was obtained by immunising four guinea pigs with the antigenic peptide [C]-PQNFVT-[pS]-QTTNV, where C represents the C-terminus of the peptide and pS is a phosphorylated serine residue. The α-pS298 antibody was purified in a similar manner to the α-pT318 antibody.

### Western blot analysis

Protein extraction and Western blot analysis of Hop1 were carried as previously described [15]. Western blot analysis of Mek1-3HA was carried out using 7.5% acrylamide gels containing 200μM of MnCl_2_ and 4μM of PhosTag (AAL-107; NARD Institute, Amagasaki, Japan). A mouse monoclonal anti-HA antibody was used for detection of Mek1-HA as previously described [6].

### Cytology

The preparation of meiotic nuclear spreads and immunofluorescence analysis were carried out as previously described [6]. The secondary antibodies used to detect the α-pT318 and α-pS298 phospho-specific antibodies were chicken anti-rabbit Alexa-594 [Invitrogen] and goat anti-guinea pig Alexa-594 [Invitrogen], respectively.

## Supporting Information

S1 Fig Effects of temperature and *hop1-S298A* on spore viability and steady state Hop1 protein level. **A, B**. Effects of *hop1-S298A* and *hop1-T318A* on Hop1-S298 or Hop1-T318 phosphorylation during *DMC1* or *dmc1Δ* meiosis at 23°C meiosis. Representation of the relative signals obtained from the quantification of the entire signal detected by western blot in A B using the anti-Hop1, anti-pT318, and anti-pS298 antibodies. **C**. Homozygous diploids of *HOP1* and *hop1-S298A* were incubated on SPM plate at the indicated temperature for either one (30°C, 33°C, 36°C) or two days (18°C, 23°C). Tetrads were dissected on YPD plates and incubated at 30°C. The images were taken following 2 day incubation. **D**. Temperature sensitivity of *hop1-S298* is not associated with a notable effect on protein stability or phosphorylation. Strains of indicated genotypes were taken through synchronous meiosis at 23°C or 33°C. Samples were collected at the indicated time points and subjected to Western Blot analysis using polyclonal antibodies to Hop1. Positions of unphosphorylated or phosphorylated Hop1 species are as indicated. Shown below each Western blot image is the corresponding ponceau staining gel as a loading control. *hop1-S298A*: non phosphorylatable allele, *hop1-S298D*: phospho-mimetic allele, *hop1-S298x2*: an allele containing two tandem copies of *hop1-S298A*.
(TIF)

S2 Fig Extent of Mek1 phosphorylation in a *dmc1Δ* background correlates with the robustness of *dmc1Δ* arrest. Homozygous diploids of indicated genotypes were taken through synchronous meiosis at 23°C. Samples were collected at the indicated time points and subjected to Western Blot analysis using anti-HA antibody for detection of Mek1-HA. Positions of unphosphorylated or phosphorylated Mek1-HA species are as indicated. Shown below are sporulation efficiency in each culture and quantification analysis of the Western images, where the signal in the ‘pMek1-HA’ region in each lane is divided by the total signal (‘pMek1-HA’+ ‘Mek1-HA’) in the corresponding lane.
(TIF)

S3 Fig Genetic interaction between *hop1-S298A* and *mek1-S320A*. Homozygous diploids of indicated genotypes were incubated on SPM plates at 18°C, 30°C, and 36°C for one (30°C, 36°C) or two days (18°C). Tetrads were dissected on YPD plates and incubated at 30°C for two days. Spore viability was calculated as the number of visible spore colonies over the total number of spores dissected. For each strain, at least 160 spores were analysed.
(TIF)

S1 Table Yeast strains used in the current study. All strains are homozygous diploids of the SK1 background of *S*. *cerevisiae*. All strains except HTY2091-2092 and APY67-68/70 were derived from JCY448 (*ho*::*LYS2/ho*::*LYS2*, *ura3/ura3*, *leu2*::*hisG/leu2*::*hisG*, *hop1Δ*::*LEU2/hop1Δ*::*LEU2) (Carballo et al*. *2008); from JCY511 (ho*::*LYS2/ho*::*LYS2*, *ura3/ura3*, *leu2*::*hisG/leu2*::*hisG*, *hop1Δ*::*LEU2/ hop1Δ*::*LEU2 dmc1Δ*::*KanMX4/ dmc1Δ*::*KanMX4*) (Carballo et al. 2008); and JCY614 (*ho*::*LYS2/ho*::*LYS2*, *ura3/ura3*, *leu2*::*hisG/leu2*::*hisG*, *hop1Δ*::*LEU2/hop1Δ*::*LEU2*, *MEK1-3HA*::*URA3/ MEK1-3HA*::*URA3*) (Carballo et al. 2008). HTY2091-HTY2092 are derived from TBR2091 and TBR2092 respectively from (Tsubouchi and Roeder, 2006). APY68-69 were derived from HTY2091 and APY67 was derived from HTY2092.
(PDF)

S2 Table Supplementary data tables. Supplementary data tables showing the values plotted in the graphs from Figs 1D and 1E, 3A, 3B and 3F, 4B–4G, and S1A and S1B, and S2 Figs.
(XLSX)
