# Correction: Essential and Checkpoint Functions of Budding Yeast ATM and ATR during Meiotic Prophase Are Facilitated by Differential Phosphorylation of a Meiotic Adaptor Protein, Hop1

**DOI:** 10.1371/journal.pone.0154170

**Published:** 2016-04-18

**Authors:** Ana Penedos, Anthony L. Johnson, Emily Strong, Alastair S. Goldman, Jesús A. Carballo, Rita S. Cha

In the section “Generation of phospho-specific Hop1 antibodies” of the Materials and Methods, we made several mistakes when indicating the sequence of the peptides used for the generation of antibodies. Please view the correct paragraph below.

## Generation of phospho-specific Hop1 antibodies

Polyclonal antibodies against the Hop1 phospho-T318 and phospho-S298 were obtained as following: The α-pT318 polyclonal antibody [Cambridge Research Biochemicals] was obtained by immunising two rabbits with the antigenic [C]-Ahx-ASIQP-[pT]-QFVSN where C is a cysteine residue added at the N-terminus of the peptide, Ahx is aminohexanoicacid and pT is a phosphorylated threonine residue. Upon bleeding, antibodies were purified through two affinity columns (each followed by a purification pass), the first adsorbing antibodies that bind to non-phosphorylated peptides and the second adsorbing the phospho-specific antibodies to pT318. The specificity of the antibody was tested using ELISA (enzyme-linked immunosorbent assay) analysis. The polyclonal phospho-specific antibody against phosphorylated serine residue 298 [Eurogentec] was obtained by immunising four guinea pigs with the antigenic peptide [C]-PQNFVT-[pS]-QTTNV, where C represents a cysteine added at the N-terminus of the peptide and pS is a phosphorylated serine residue. The α-pS298 antibody was purified in a similar manner to the α-pT318 antibody.
