# Supplementary figures and images for: Essential and Checkpoint Functions of Budding Yeast ATM and ATR during Meiotic Prophase Are Facilitated by Differential Phosphorylation of a Meiotic Adaptor Protein, Hop1
Source: PLoS One. 2015 Jul 30;10(7):e0134297. doi: 10.1371/journal.pone.0134297 (PMC4520594; doi:10.1371/journal.pone.0134297)

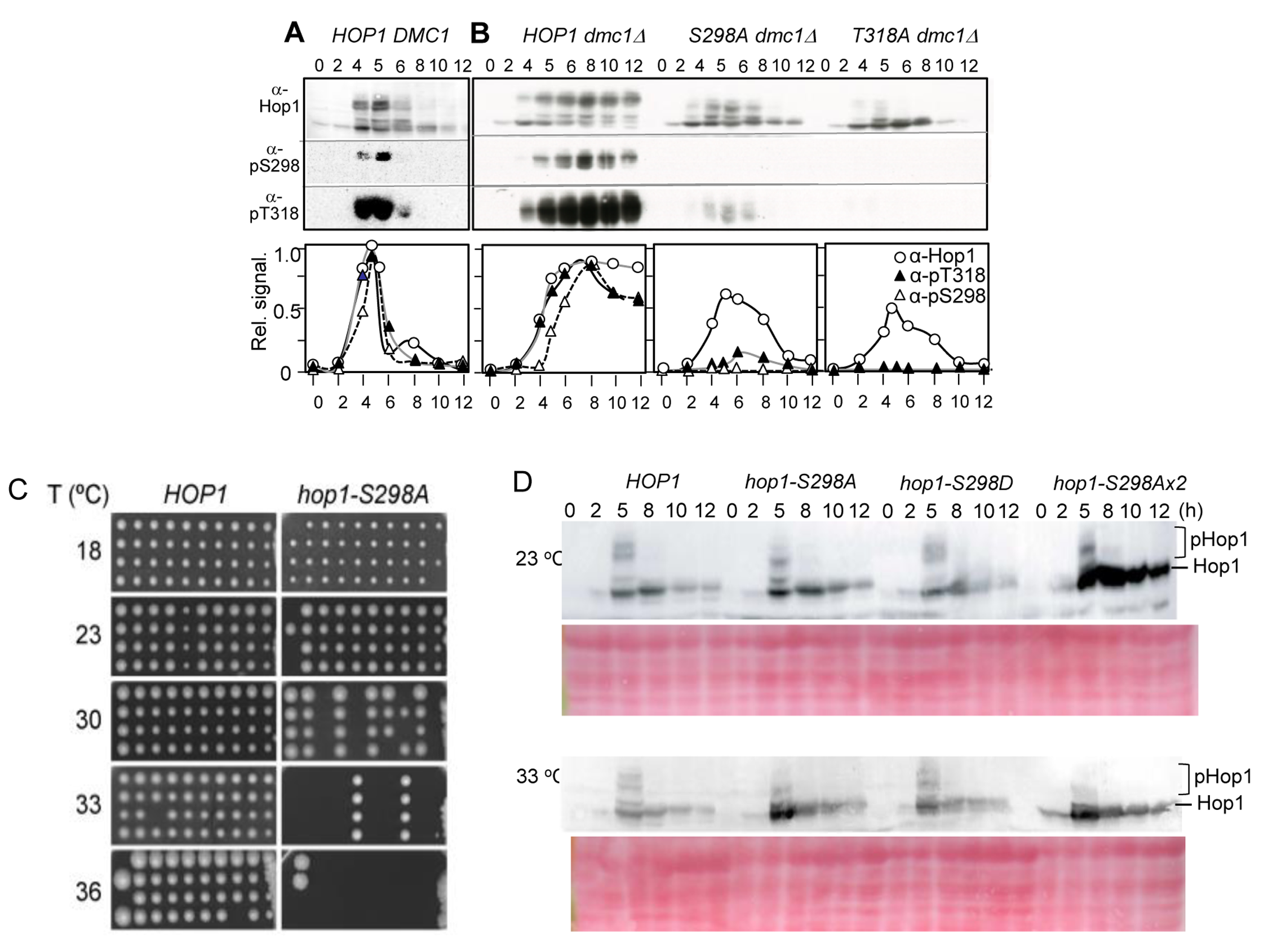

Supplement: S1 Fig — A, B. Effects of hop1-S298A and hop1-T318A on Hop1-S298 or Hop1-T318 phosphorylation during DMC1 or dmc1Δ meiosis at 23°C meiosis. Representation of the relative signals obtained from the quantification of the entire signal detected by western blot in A B using the anti-Hop1, anti-pT318, and anti-pS298 antibodies. C. Homozygous diploids of HOP1 and hop1-S298A were incubated on SPM plate at the indicated temperature for either one (30°C, 33°C, 36°C) or two days (18°C, 23°C). Tetrads were dissected on YPD plates and incubated at 30°C. The images were taken following 2 day incubation. D. Temperature sensitivity of hop1-S298 is not associated with a notable effect on protein stability or phosphorylation. Strains of indicated genotypes were taken through synchronous meiosis at 23°C or 33°C. Samples were collected at the indicated time points and subjected to Western Blot analysis using polyclonal antibodies to Hop1. Positions of unphosphorylated or phosphorylated Hop1 species are as indicated. Shown below each Western blot image is the corresponding ponceau staining gel as a loading control. hop1-S298A: non phosphorylatable allele, hop1-S298D: phospho-mimetic allele, hop1-S298x2: an allele containing two tandem copies of hop1-S298A. (TIF) [file pone.0134297.s001.tif]

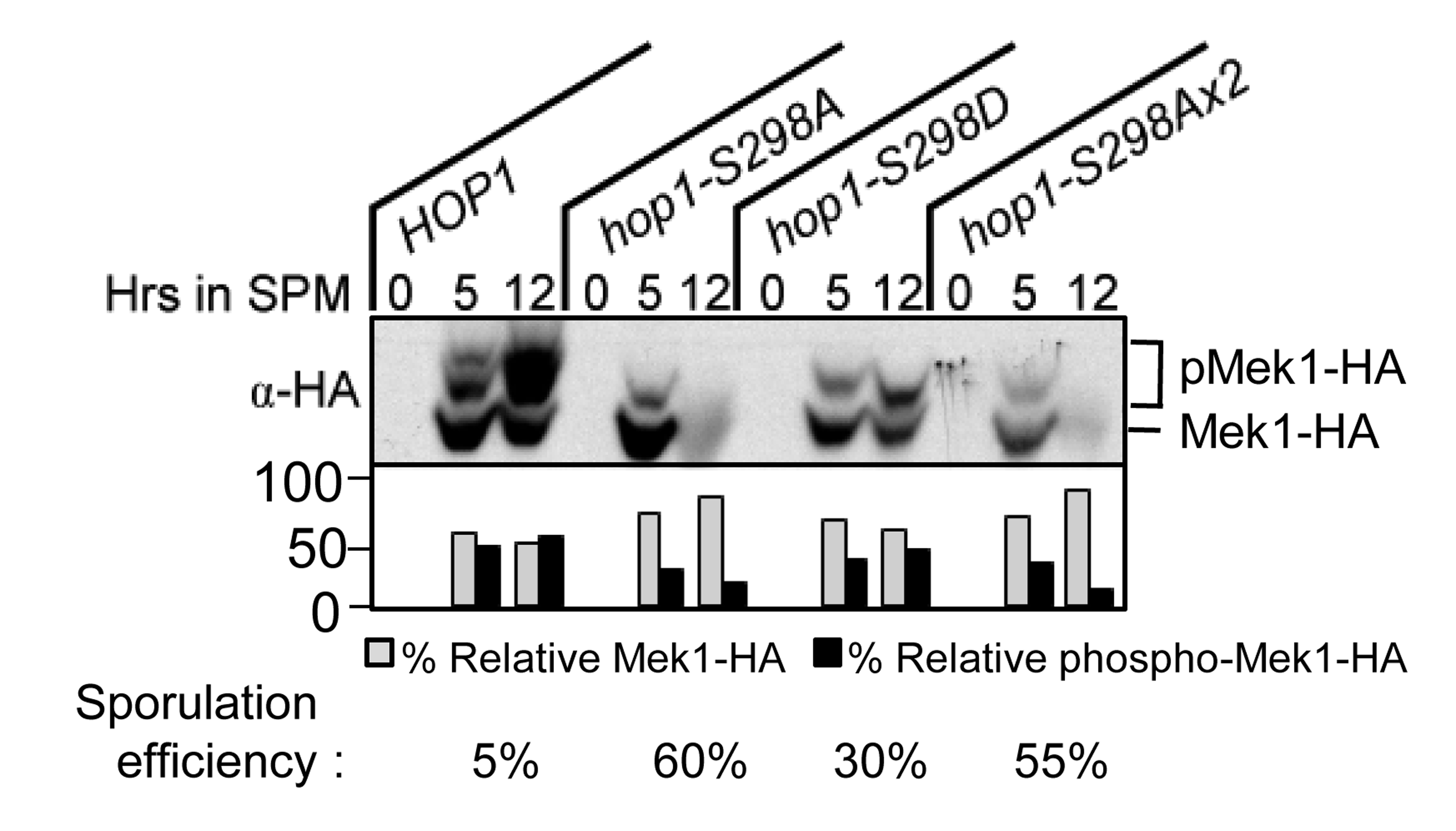

Supplement: S2 Fig — Homozygous diploids of indicated genotypes were taken through synchronous meiosis at 23°C. Samples were collected at the indicated time points and subjected to Western Blot analysis using anti-HA antibody for detection of Mek1-HA. Positions of unphosphorylated or phosphorylated Mek1-HA species are as indicated. Shown below are sporulation efficiency in each culture and quantification analysis of the Western images, where the signal in the ‘pMek1-HA’ region in each lane is divided by the total signal (‘pMek1-HA’+ ‘Mek1-HA’) in the corresponding lane. (TIF) [file pone.0134297.s002.tif]

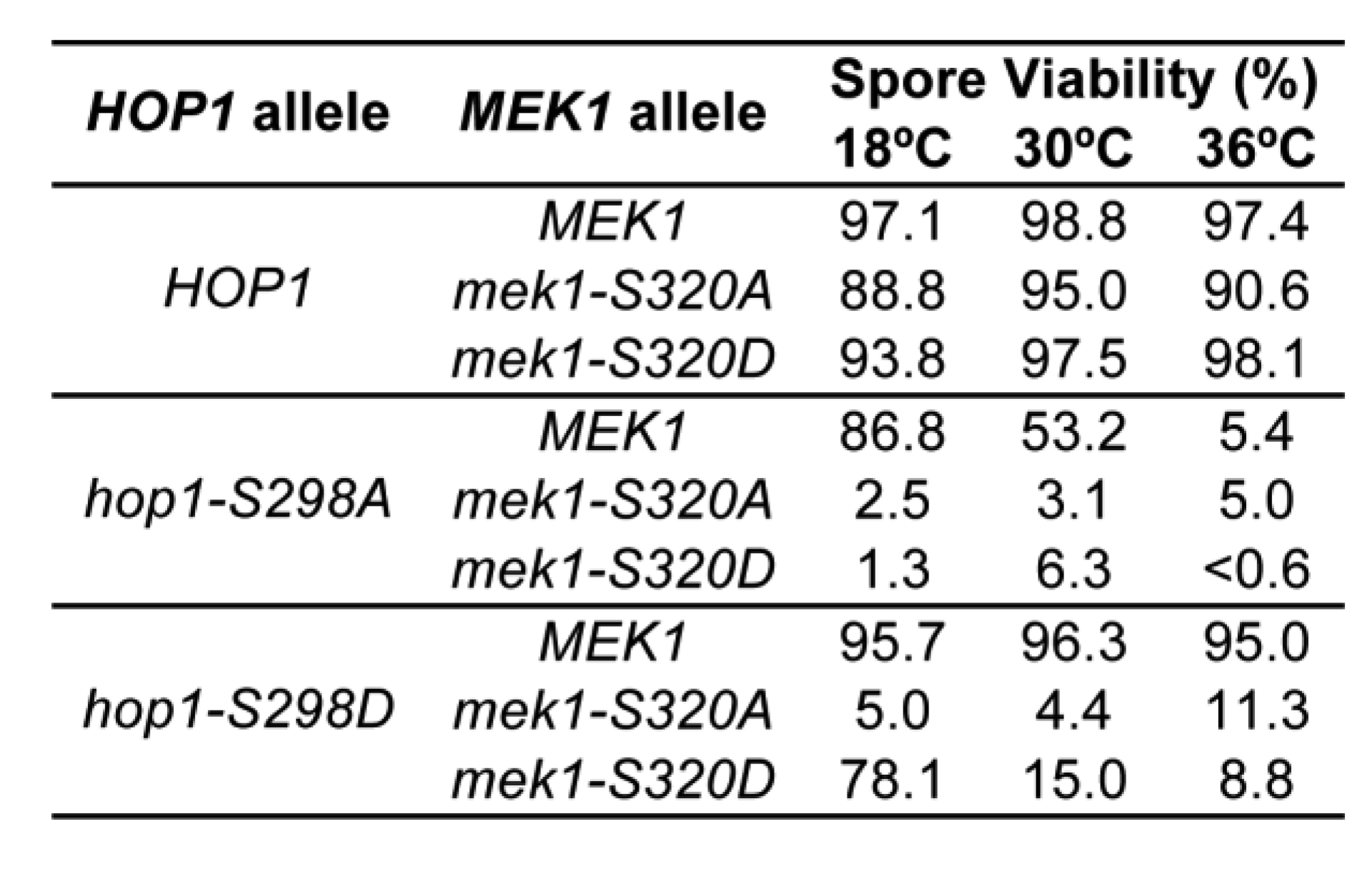

Supplement: S3 Fig — Homozygous diploids of indicated genotypes were incubated on SPM plates at 18°C, 30°C, and 36°C for one (30°C, 36°C) or two days (18°C). Tetrads were dissected on YPD plates and incubated at 30°C for two days. Spore viability was calculated as the number of visible spore colonies over the total number of spores dissected. For each strain, at least 160 spores were analysed. (TIF) [file pone.0134297.s003.tif]
