# Supplementary material for: Essential and Checkpoint Functions of Budding Yeast ATM and ATR during Meiotic Prophase Are Facilitated by Differential Phosphorylation of a Meiotic Adaptor Protein, Hop1
Source: PLoS One. 2015 Jul 30;10(7):e0134297. doi: 10.1371/journal.pone.0134297 (PMC4520594; doi:10.1371/journal.pone.0134297)
Supplement: S1 Table — All strains are homozygous diploids of the SK1 background of S. cerevisiae. All strains except HTY2091-2092 and APY67-68/70 were derived from JCY448 (ho::LYS2/ho::LYS2, ura3/ura3, leu2::hisG/leu2::hisG, hop1Δ::LEU2/hop1Δ::LEU2) (Carballo et al. 2008); from JCY511 (ho::LYS2/ho::LYS2, ura3/ura3, leu2::hisG/leu2::hisG, hop1Δ::LEU2/ hop1Δ::LEU2 dmc1Δ::KanMX4/ dmc1Δ::KanMX4) (Carballo et al. 2008); and JCY614 (ho::LYS2/ho::LYS2, ura3/ura3, leu2::hisG/leu2::hisG, hop1Δ::LEU2/hop1Δ::LEU2, MEK1-3HA::URA3/ MEK1-3HA::URA3) (Carballo et al. 2008). HTY2091-HTY2092 are derived from TBR2091 and TBR2092 respectively from (Tsubouchi and Roeder, 2006). APY68-69 were derived from HTY2091 and APY67 was derived from HTY2092. (PDF) [file pone.0134297.s004.pdf]

Strain	Genotype
APY1-2	*hop1Δ::LEU2/”, ura3::HOP1::URA3/”*
JCY555-556	*hop1Δ::LEU2/”, ura3:: hop1*^{SCD}*::URA3/”*
JCY559-560	*hop1Δ::LEU2/”, ura3::hop1-S298A::URA3/”*
APY172-173	*hop1Δ::LEU2/”, ura3::hop1-S298A::URA3x2/”*
APY214-215	*hop1Δ::LEU2/”, ura3::hop1-S298D::URA3/”*
JCY604/610	*hop1Δ::LEU2/”, ura3::hop1-S311A::URA3/”*
JCY553-554	*hop1Δ::LEU2/”, ura3::hop1-T318A::URA3/”*
JCY593-594	*hop1Δ::LEU2/”, ura3::HOP1::URA3/”, dmc1Δ::KanMX4/”*
JCY587-588	*hop1Δ::LEU2/”, ura3:: hop1*^{SCD}*::URA3/”, dmc1Δ::KanMX4/”*
JCY591-592	*hop1Δ::LEU2/”, ura3::hop1-S298A::URA3/”, dmc1Δ::KanMX4/”*
APY209-210	*hop1Δ::LEU2/”, ura3::hop1-S298A::URA3x2/”, dmc1Δ::KanMX4/”*
APY199-200	*hop1Δ::LEU2/”, ura3::hop1-S298D::URA3/”, dmc1Δ::KanMX4/”*
APY15-16	*hop1Δ::LEU2/”, ura3::hop1-S311A::URA3/”, dmc1Δ::KanMX4/”*
JCY585	*hop1Δ::LEU2/”, ura3::hop1-T318A::URA3/”, dmc1Δ::KanMX4/”*
HTY2091	*hed1Δ::hphMX4/”*
APY68/70	*hop1Δ::LEU2/”, ura3::hop1-S298A::URA3/”, hed1Δ::hphMX4/”*
HTY2092	*dmc1Δ::KanMX4/”, hed1Δ::hphMX4/”*
APY67	*hop1Δ::LEU2/”, ura3::hop1-S298A::URA3/”, dmc1Δ::KanMX4/”, hed1Δ::hphMX4/”*
JCY623	*hop1Δ::LEU2/”, ura3::HOP1::URA3/”, rad50S::URA3/”*
APY50	*hop1Δ::LEU2/”, ura3::hop1-S298A::URA3/”, rad50S::URA3/”*
APY83	*hop1Δ::LEU2/”, ura3::HOP1::URA3/”, MEK1-3HA::URA3/”*
APY85	*hop1Δ::LEU2/”, ura3::hop1-S298A::URA3/”, MEK1-3HA::URA3/”*
APY405	*hop1Δ::LEU2/”, ura3::hop1-S298A::URA3x2/”, MEK1-3HA::URA3/”*
APY404	*hop1Δ::LEU2/”, ura3::hop1-S298D::URA3/”, MEK1-3HA::URA3/”*
APY370-371	*hop1Δ::LEU2/”, ura3::hop1-T318A::URA3/”, MEK1-3HA::URA3/”*
APY134	*hop1Δ::LEU2/”, ura3::HOP1::URA3/”, MEK1-3HA::URA3/”, dmc1Δ::KanMX4/”*
APY32-33	*hop1Δ::LEU2/”, ura3::hop1-S298A::URA3/”, MEK1-3HA::URA3/”, dmc1Δ::KanMX4/”*
APY422	*hop1Δ::LEU2/”, ura3::hop1-S298A::URA3x2/”, MEK1-3HA::URA3/”, dmc1Δ::KanMX4/”*
APY420	*hop1Δ::LEU2/”, ura3::hop1-S298D::URA3/”, MEK1-3HA::URA3/”, dmc1Δ::KanMX4/”*
JCY573	*hop1Δ::LEU2/”, ura3::hop1-T318A::URA3/”, MEK1-3HA::URA3/”, dmc1Δ::KanMX4/”*
